# Anaphylactic Food Allergy After Roux-en-Y Gastric Bypass

**DOI:** 10.7759/cureus.17710

**Published:** 2021-09-04

**Authors:** James Kamau, Shannon Kearny, Aaron Jaworek, Richard Snyder, Maher El Chaar

**Affiliations:** 1 Internal Medicine, St. Luke’s University Health Network, Easton, USA; 2 Allergy and Immunology, St. Luke’s University Health Network, Bethlehem, USA; 3 Otolaryngology, St. Luke’s University Health Network, Bethlehem, USA; 4 Bariatric Surgery, St. Luke’s University Health Network, Bethlehem, USA

**Keywords:** food allergy, food intolerance, allergy and anaphylaxis, gastric bypass surgery, allergy

## Abstract

The prevalence of obesity in the United States is projected to increase as high as 85% by 2030. Weight loss is associated with improved morbidity and mortality outcomes. Roux-en-Y gastric bypass (RYGB) is an effective procedure recommended for individuals with morbid obesity for weight loss. We report the case of a patient who developed worsening food allergic reactions after RYGB surgery that progressed to an anaphylactic reaction. A 36-year-old female developed an anaphylactic reaction to an ingredient in guacamole eight years after RYGB surgery. Prior to the surgery, she had symptoms consistent with oral allergy syndrome. After the gastric bypass, however, she experienced worsening symptoms. On this occasion, she developed throat tightness prompting a visit to the emergency department where she required emergent intubation for airway protection. Blood testing to assess for an immunoglobin E-mediated allergy to common foods was negative. Despite the negative test, the allergist maintained a high suspicion for the progression of food-pollen syndrome following gastric bypass. Disruption of protein digestion from stomach bypass surgery may result in dietary proteins large enough to elicit immune responses being presented to the immune-rich intestinal mucosa. Additional consideration should be given to patients with a preexisting history of food allergic reactions undergoing RGYB surgery.

## Introduction

The prevalence of obesity in the United States was 42.4% in 2017-2018 [1]. This has been projected to increase as high as 85% by 2030 including adults who are classified as overweight or obese [2]. Obesity is associated with an increased risk of depression, type 2 diabetes, obstructive sleep apnea, cardiovascular disease, and malignancy [3]. Roux-en-Y gastric bypass (RYGB) is a safe and effective procedure recommended for individuals with morbid obesity for weight loss [4]. There have been reports of the development of food allergy after RYGB due to disruption of the digestive process as a consequence of this procedure [5]. Food sensitivity occurs because of an inappropriate immune response to dietary antigens [6]. Here, we report the case of a patient who developed worsening food allergic reactions after RYGB surgery that progressed to an anaphylactic reaction.

## Case presentation

A 36-year-old female with a surgical history of RYGB presented to the emergency department (ED) with urticaria, tongue swelling, and voice changes. She had stable vital signs but appeared anxious. She reported that prior to gastric bypass surgery, she would develop itching in her mouth and throat whenever she ate raw fruits and vegetables, consistent with oral allergy syndrome. After the gastric bypass, however, she experienced nausea and vomiting whenever she ate raw fruits and vegetables, including guacamole. On this occasion, she developed itching in her mouth and throat approximately 20 minutes after she ate guacamole. This progressed to eyelid swelling and throat tightness prompting her to go to the ED. On examination, she had upper and lower lip swelling, mild tongue swelling, and a grossly swollen uvula that was in contact with the tongue but no stridor. Her skin was notable for hives on the lower back and upper arms. The rest of her physical exam was unremarkable.

Given the physical examination findings, she required immediate administration of epinephrine, methylprednisolone, famotidine, and diphenhydramine. She was reassessed approximately 20 minutes after medical treatment and was found to have worsening symptoms including trouble swallowing. Her examination was now significant for worsening oral mucosal swelling involving the entire soft palate with the inability to see the posterior oropharynx. She was subsequently emergently intubated for airway protection and admitted to the intensive care unit. She was extubated two days later.

She underwent blood testing to assess for an immunoglobin E (IgE)-mediated allergy to common foods, which was negative. Despite this, the allergy team maintained a high suspicion for progression of food-pollen syndrome with increased absorption of dietary antigens following gastric bypass. She continues to follow up with an allergy specialist and has had no further issues with avocado or any other specific food avoidance.

## Discussion

Food allergy is an IgE-mediated dysregulation of the immune system related to the body’s defense against enteral pathogens. Symptoms associated with this diagnosis can range from mild to fatal, namely, oral tingling, skin rash, nausea, vomiting, abdominal pain, diarrhea, and anaphylaxis. Food allergy is common with a prevalence of up to 10% of US adults [7]. In general, cows’ milk, eggs, peanuts, tree nuts, fish, shellfish, wheat, and soy account for the majority of food-related allergic reactions [8].

Protein digestion has a profound effect on the development of food allergies [9]. The acidic environment of the stomach is critical to food protein digestion. The activity of pepsin, a proteolytic enzyme found in the stomach, is optimal between pH 1.8 and 3.2 and decreases with increasing gastric pH [10]. Pepsin initiates the protein digestion process in the stomach. These partially digested proteins are further degraded in the intestines by proteases and peptidases produced by the pancreas into amino acids that are too small to stimulate an immune response [11].

Any disruption to this process such as acid suppression or a gastric bypass may result in dietary proteins large enough to elicit immune responses being presented to the immune-rich intestinal mucosa [12]. This was demonstrated in murine experiments where dietary proteins that were encapsulated to prevent degradation during gastrointestinal transit were shown to induce oral allergy in murine models with previously established oral tolerance [13]. The same effect was seen in patients treated with acid-suppressant medications whose relative risk of developing food-specific IgE after antacid therapy was 10.5 [14].

RYGB is a surgical procedure that involves transection of the superior portion of the stomach to create a small pouch, which is anastomosed to the proximal jejunal segment, essentially bypassing the rest of the stomach, duodenum, and part of the jejunum. RYGB induces weight loss by limiting food intake. The increased gastric transit time [15] and lower gastric acidity [16] as a result of the procedure may interfere with protein digestion. In fact, a study found that about 77% of the patients who underwent an RYGB developed increased sensitization toward tested food, further supporting the gastric gatekeeping function with regards to oral tolerance [5].

Gastric bypass surgery has also been associated with an increased incidence of celiac disease, another immune-related disease caused by gluten sensitivity [17]. However, the pathogenesis of celiac disease differs from that of food allergy. Celiac disease is an autoimmune condition where the immune system is directed to one’s own body rather than against foreign substances. Celiac disease also utilizes a different branch of the immune response as opposed to IgE in allergic reactions. However, much like food allergy, a dietary protein (gluten) triggers this immune-mediated inflammatory response. Decreased degradation of this protein is seen in individuals with active celiac disease, which may elicit an autoimmune response in susceptible patients [18]. The use of specific endoproteases and endopeptidases with gastric activity in in vitro and in vivo studies using animal models has been shown to increase the safe threshold of ingested gluten [19]. The unmasking phenomenon observed in the development of celiac disease in patients after gastric bypass is likely due to the interference of protein digestion in susceptible individuals.

More studies are warranted to investigate the association between RYGB surgery and food sensitivity. For now, it may be prudent to refer symptomatic patients who have undergone RYGB surgery to an allergy specialist for further evaluation.

## Conclusions

Additional consideration should be applied to adult patients with a preexisting history of food allergic reactions undergoing gastric bypass surgeries such as RYGB. When identified, preoperative counseling in this patient population may be prudent. These patients may also require close follow-up with an allergy specialist and an epinephrine autoinjector for emergent situations.
